# Correcting Class III Malocclusion Using Multiloop Edgewise Archwire (MEAW) Combines Temporary Anchorage Devices (TADs) With Modified Class III Elastics for Camouflage Treatment of in Adolescent: A Case Report

**DOI:** 10.1155/crid/5546633

**Published:** 2024-11-19

**Authors:** Lam Nguyen Le, Thao Thi Do, Khanh Phuong Vu Le

**Affiliations:** ^1^Department of Pediatrics and Orthodontics, Faculty Odonto-Stomatology, Can Tho University of Medicine and Pharmacy, Can Tho City, Vietnam; ^2^Department of Oral Pathology and Periodontology, Faculty Odonto-Stomatology, Can Tho University of Medicine and Pharmacy, Can Tho City, Vietnam; ^3^Faculty Odonto-Stomatology, Can Tho University of Medicine and Pharmacy, Can Tho City, Vietnam

**Keywords:** case report, Class III elastics, miniscrew, multiloop edgewise archwire (MEAW), nonsurgery, orthodontics, temporary anchorage devices (TADs)

## Abstract

**Introduction:** One of the most difficult types of malocclusion to cure is Class III malocclusion. Maxillary protraction is a common component of orthodontic/surgical treatments for Class III individuals since maxillary retrognathia is the primary factor contributing to their condition.

**Objective:** A Vietnamese girl of 16 years old presented with severe symptoms of anterior crossbite and Class III malocclusion of the skeletal and dental structures. Among the dental characteristics were a prognathic mandible, retrognathic maxilla, and proclined lower incisors. The medical history revealed no abnormalities.

**Methods:** Since the patient refused to undergo orthognathic surgery, we suggested using multiloop edgewise archwire (MEAW), which combines temporary anchorage devices (TADs) with modified Class III elastics for camouflage treatment of Class III malocclusion.

**Results:** This case illustrated the use of TADs. MEAW combines TADs with modified Class III elastics for camouflage therapy. It is a potentially effective treatment option for adolescents diagnosed with Class III skeletal malocclusion.

## 1. Introduction

Orthognathic surgery or orthodontic camouflage treatment may methodically treat skeletal Class III malocclusion, which is characterized by the asymmetry of the mandibular and maxilla. Surgical interventions are advised in extreme instances to rectify skeletal and dental irregularities, enhance face aesthetics, and achieve facial symmetry [1, 2]. Camouflage may be considered an acceptable option for treatment. The goal of this approach is to minimize the visibility of dental and skeletal variations to the greatest degree possible while considering the patient's facial appearance and functional issues. At times, achieving this goal may be challenging due to insufficient anchoring, which is critical for the successful outcome of orthodontic treatment [3, 4]. Using the multiloop edgewise archwire (MEAW) technique and Class III elastics effectively address the position of the mandibular, restores the occlusal plane, and modifies the breadth of both dental arches. The posterior teeth exhibit increased torque and contribute to the correction of Class III discrepancies [5–7]. The protrusion of the maxillary teeth may cause the mandible to rotate in a clockwise direction, leading to an increase in the height of the lower front teeth and a decrease in the depth of the bite. This phenomenon will have advantageous implications in the management of individuals exhibiting a mandible with a low flat angle and a deep bite, as it facilitates the rectification of the misalignment of the teeth and concurrently diminishes the protrusion of the chin. A significant mandibular plane angle and heightened anteroinferior height were skeletal Class III malocclusion anatomical characteristics [8]. The protrusion of the maxillary teeth causes the mandible to rotate in a clockwise direction, resulting in an unwanted increase in the height of the front and lower parts of the jaw. The maxillary anterior teeth are already tilted because of dentoalveolar compensation in most skeletal Class III patients. Class III elastics could make this tilt even worse. This can potentially compromise the patient's profile and aesthetic treatment stability outcomes [9]. Maintaining a well-compensated occlusion for the back teeth is critical when considering camouflage orthodontic treatment for facial skeletal asymmetry and Class III malocclusion. An unstable bite, such as a scissor bite or crossbite, may develop when orthodontic treatment changes the angle of the compensatory bite [5].

An asymmetrical face, transverse discrepancy, and skeletal Class III malocclusion were the individual's diagnoses in this case report. The goal of this report was to suggest that MEAW combines TADs with modified Class III elastics for camouflage treatment as a new way to solve Class III malocclusions.

## 2. Diagnosis and Etiology

**Figure 1:**
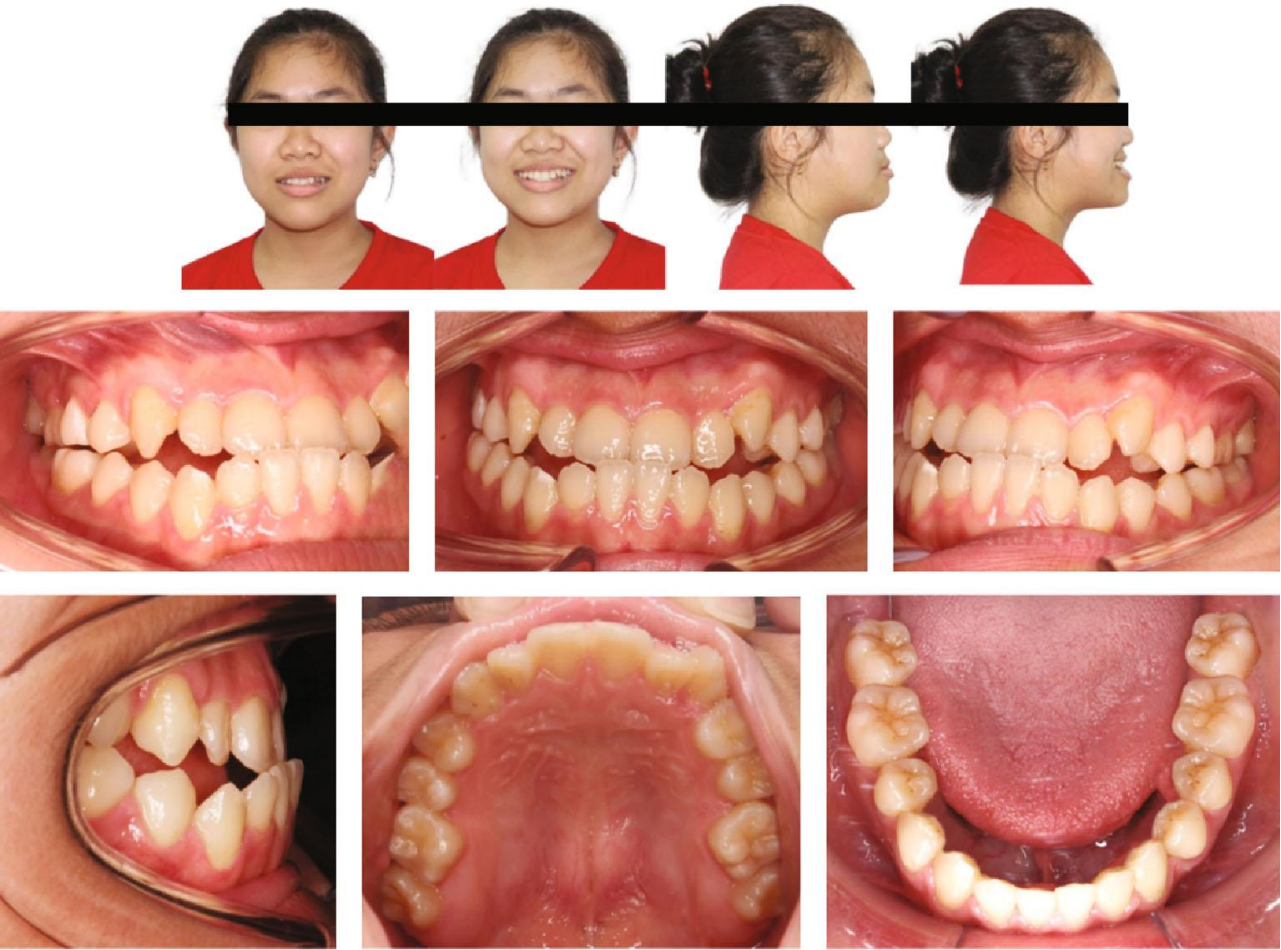
Pretreatment facial and intraoral photographs (16-year-old female).

**Figure 2:**
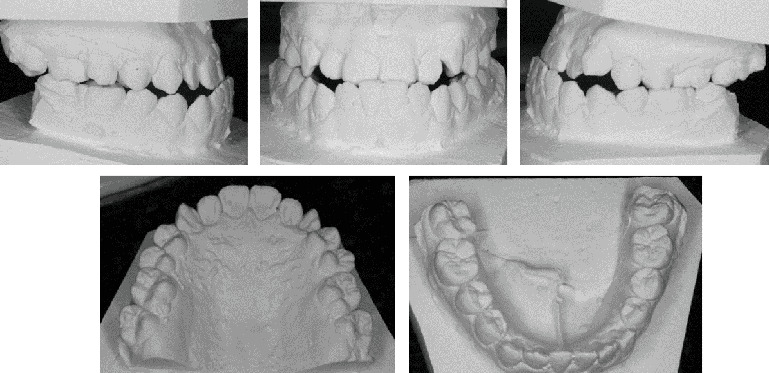
The study models (casts) of pretreatment.

A Vietnamese girl of 16 years old presented with severe symptoms of anterior crossbite and Class III malocclusion of the skeletal and dental structures. The medical history revealed no abnormalities. The frontal view revealed a flatter, longer lower face with a retracted upper lip. Facial asymmetry was significant in the smile arc. The facial midline and the upper midline were coincidental. A prognathic mandible, retrognathic maxilla, and proclined lower incisors were the dental characteristics. A Class III relationship existed between the canines and molars, and the maxillary arch was symmetrical, whereas the mandibular arch was asymmetrical (Figure 1). Digital caliper space analysis revealed moderate crowding in the upper arch (Figure 2).

**Figure 3:**
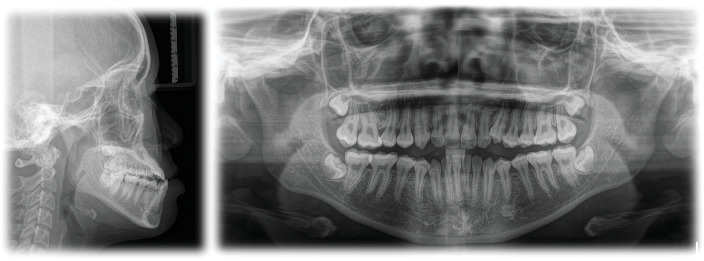
Pretreatment lateral cephalogram and panoramic radiograph.

The patient had a skeletal Class III malocclusal with facial asymmetry and transverse discrepancy, which is what Vietnamese standards say. This was shown by the lateral cephalometric radiography and its correlation (Table 1). The upper and lower dentition minor presented crowding (−2.5 mm and −0.5 mm). According to the panoramic radiograph, the condyles seemed to be well balanced. No skeletal diseases and all the following were normal: tooth morphology, bone level, temporomandibular joint, and maxillary sinus. The panoramic examination turned up nothing else noteworthy, except for the growing third molars (Figure 3).

### 2.1. Treatment Goals

The objectives of the treatment were as follows: (1) to create good occlusion; (2) to address horizontal discrepancies; (3) to enhance the inclination of the compensated teeth; (4) to address discrepancies between the teeth; (5) to preserve periodontal health; and (6) to develop an aesthetic profile. In addition, steady observation of skeletal growth was required.

### 2.2. Treatment Plan

Retracting the mandibular and correcting the anterior crossbite were the goals of this camouflage treatment for solving the Class III malocclusal. To alleviate crowding and retracting the lower lip, MEAW combines TADs with modified Class III elastics for camouflage treatment.

### 2.3. Treatment Alternatives

To achieve the treatment goal, there were three possibilities reviewed and discussed with the patient.

Option 1 combines orthopedic surgery and orthodontic treatment, utilizing LeFort I osteotomies of the upper progression and bilateral longitudinal osteotomies of the mandibular recession. The advantages of this plan include an improved facial profile and improved congestion.

Option 2 involved orthodontic treatment, which involved extracting the first four premolars to conceal the concave appearance and correct the blockage. The plan entailed inclining the upper teeth toward the lips. Although the thin trabecular bone may increase the risk of excessive lingual movement of the lower incisors, it was possible to enhance the anterior teeth. Additionally, this plan could create a facial appearance that deteriorates with a prominent chin.

Option 3 is nonextraction orthodontic treatment. The distal movement of the mandibular arch using a MEAW technique, combined with maxilla TADs, was required. This plan would use Class III elastics to correct the anterior crossbite and establish a Class I molar relationship. The patient's concave face can be camouflaged.

After explaining all of these treatment plans to the patient, the third option was chosen, and informed consent was obtained. The first option (combined orthognathic surgery and orthodontic treatment) was considered an effective method for obtaining the best result. However, the patient refused the surgical approach, taking into account the higher risk of complications and cost compared to the nonsurgical options.

### 2.4. Treatment Progress

**Figure 4:**
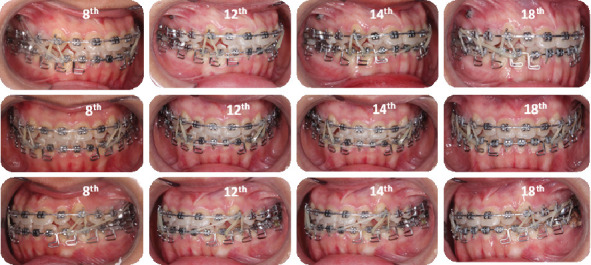
A multiloop edgewise archwire (MEAW) with tip-back bends in the lower part and elastic chains connecting the anterior teeth to miniscrews on the upper to tilt the anterior teeth of the upper toward the lips. Class III elastics (Quail, 5/16 in, 4 oz; Ormco) from miniscrews maxillary to L3s (8th month); light short triangle elastics (Quail, 3/16 in, 2 oz; Ormco) from U3s to L3s to L4s and light elastics (Quail, 5/16 in, 4 oz; Ormco) from LR3 to UL3 to correct the lower midline (12th–14th month); light short triangle Class III elastics (Quail, 3/16-in, 2 oz; Ormco) from L3s to U6s, box elastics (Fox, 1/4⁣^″^, 3.5 oz; Ormco) from U6s to U5s to L5s to L6s, and light short triangle elastics (Quail, 3/16 in, 2 oz; Ormco) from U3s to L3s to L4s (18th month).

**Figure 5:**
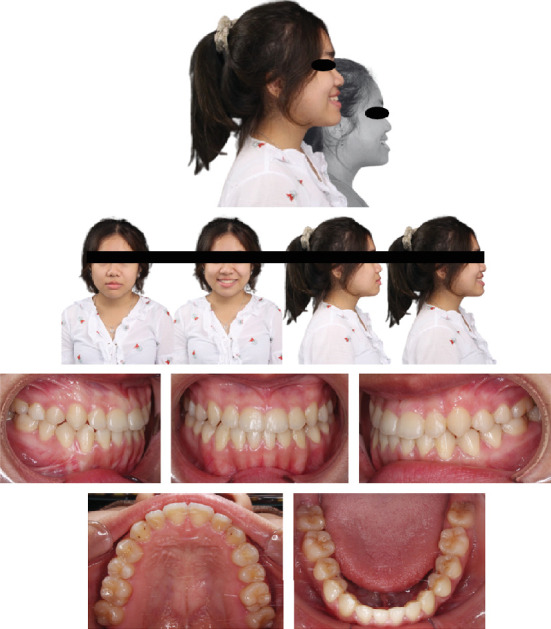
Post-treatment extraoral and intraoral photographs.

Before active orthodontic treatment, treatment began with third lower molar extractions to facilitate distal movements of the lower second and first molars. Three weeks later, 3 M Unitek (Monrovia, CA, USA) US Victory Series 0.022-in Slot Fixed Appliance Brackets (MBT −0.022 slot with standard torque) on the upper teeth with a 0.014-in CuNiTi archwire (3 M Unitek, Monrovia, CA, USA) engaged. After 3 months, 0.022-in slot Victory Series Brackets were bonded to the lower with a 0.014-in CuNiTi. The initial mechanics for both arches were 0.014-in CuNiTi archwires fitted with resin balls bonded on the ends to prevent mucosal irritation. In the 5^th^ month, both archwires were changed to 0.014 × 0.025-in NiTi. In the 8^th^ month, the leveling and alignment were completed a 0.019 × 0.025-inch stainless steel (SS) archwire was engaged in the upper, and a 0.016 × 0.022-inch TMA MEAW with a tip back bend was engaged in the lower. A temporary anchorage device (TADs), miniscrew 1.6 × 10 mm (OrthAnchorTM, Osstem, Bracket Head, Korea), was placed at the buccal region between the second premolar and first molar of the maxillary. A MEAW has a cinch back bend in the lower arch and elastic chains connecting the anterior teeth to miniscrews on the upper to tilt the anterior teeth of the upper toward the lips: Class III elastics (Quail, 5/16 in, 4 oz; Ormco) from miniscrews maxillary to L3s (8th month); light short triangle elastics (Quail, 3/16 in, 2 oz; Ormco) from U3s to L3s to L4s and light elastics (Quail, 5/16 in, 4 oz; Ormco) from LR3 to UL3 to correct the lower midline (12th–14th month); light short triangle Class III elastics (Quail, 3/16-in, 2 oz; Ormco) from L3s to U6s, box elastics (Fox, 1/4⁣^″^, 3.5 oz; Ormco) from U6s to U5s to L5s to L6s, and light short triangle elastics (Quail, 3/16 in, 2 oz; Ormco) from U3s to L3s to L4s (18th month) (Figure 4). After 28 months of active treatment, all fixed appliances were removed. The miniscrews were removed after treatment, after debonding (Figure 5).

### 2.5. Retention

A fixed retainer was positioned on the lingual surfaces from the 3^rd^ quadrant lower first premolar to the 4^th^ quadrant first premolar to stop crowding from relapse. For 6 months, the patient was told to wear the Hawley retainers full-time, and a 6-month recall appointment for a retention check.

### 2.6. Results Achieved

**Figure 6:**
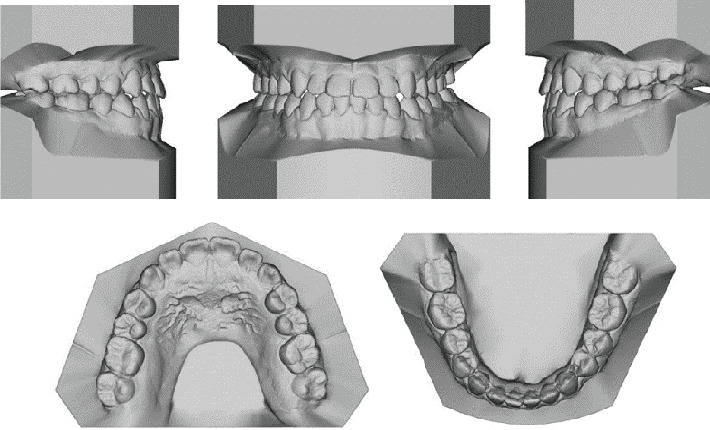
Post-treatment study models (scan).

**Figure 7:**
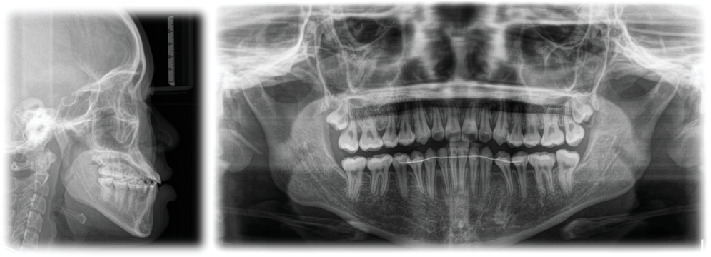
Post-treatment radiographs.

**Figure 8:**
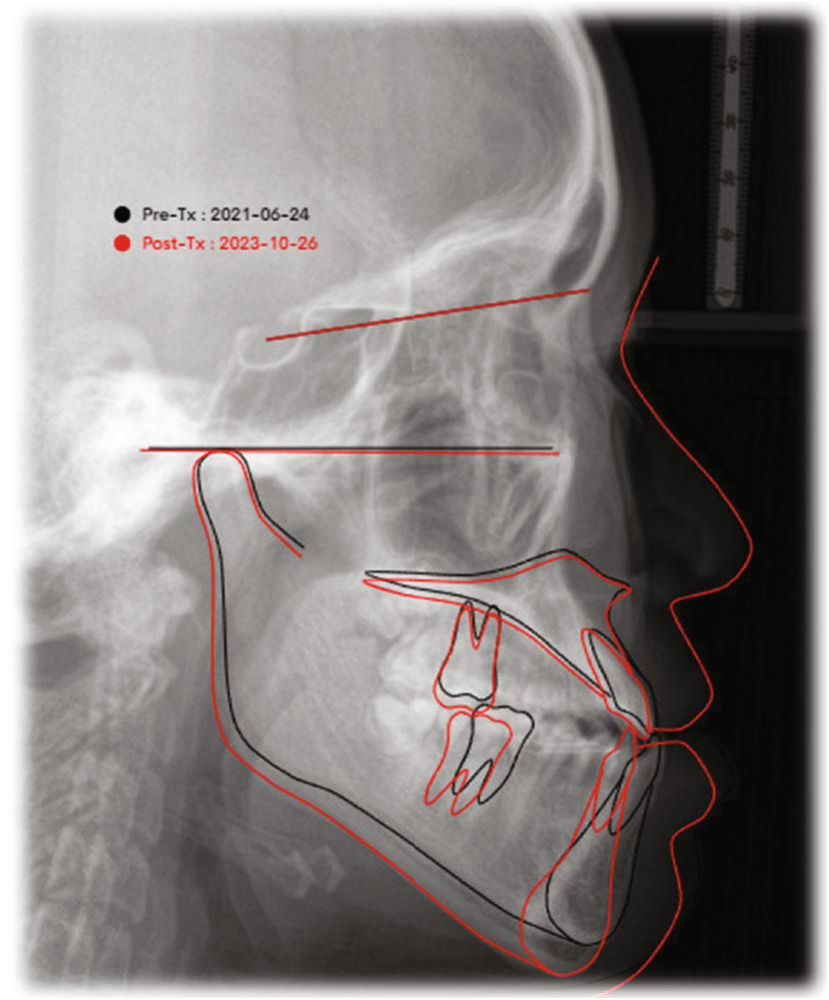
Superimposed cephalometric tracings (black: pretreatment; red: posttreatment).

Facial aesthetics and the anterior crossbite with angle Class I were significantly improved after 28 months of active treatment. The post-treatment panoramic radiograph documented acceptable root parallelism. An effective treatment was shown (Figures 6 and 7). The upper and lower midline coincided with the facial midline and improved the asymmetry of the facial (Figures 6 and 8). A positive overbite and overjet were established, accompanied by lower incisors being retracted and retroclined along with the extrusion, and the lower molars were tipped backward with mandibular distalization. The lower lip was retruded following the retraction of the anterior segments might have resulted from compensatory alveolar growth, and soft tissue changes accompanying the skeletal and dental changes might have resulted from compensatory alveolar growth concerning the vertical growth of the mandible and maxilla (Figure 7). Without orthognathic surgery, the facial profile and occlusion were satisfied. The mandibular plane angle (SN-MP) was well maintained, and the ANB angle increased with retrusion of the chin, resulting in a straight profile in superimposed lateral cephalometric tracings before and after treatment. The upper incisors were retroclined 1 mm (U1 to FH). The axial inclination of the upper incisors (U1-FH) decreased by 1° after treatment and the axial inclination of the lower incisors (L1-MP) was retroclined (Table 1).

## 3. Discussion

Orthodontists are now researching and designing biomechanical solutions to address the negative consequences of anchor tooth protrusion, mandibular rotation, and increased anterior lower facial height that occur after treatment. There was a Class III malocclusion caused by bone issues [10, 11]. An often-used approach in orthodontic camouflage treatment was the utilization of intermaxillary Class III elastics to rectify vertical disparities [12]. Class III elastics induced mesial displacement of the upper teeth and distal movement of the lower teeth, accompanied by inclination of the upper teeth and inclination of the lower molars [13, 14]. In addition, they induce the protrusion of both the upper and lower incisors, resulting in an anticlockwise rotation of the occlusal plane and an increase in facial height [15]. Nevertheless, having upper incisors that were slanted and a grin that seemed flat was considered undesirable in terms of aesthetic outcomes. The alignment and angulation of the upper incisors, as well as the angulation of the occlusal plane, played a crucial role in determining facial and smile aesthetics. To avoid these unfavorable changes, several investigations have shown the distalization of mandibular teeth using miniscrews [16, 17]. The use of miniscrews in orthodontics has been growing rapidly in the last few years [18]. The risks connected with the use of these devices are root injuries, failure, or fracture, but the technique is considered safe and predictable [19, 20].

Class III malocclusion is quite common in the Asian population, and the protrusion of the premolars is another common feature in the Oriental races. There is a common problem that orthodontists must consider whether the patient has protrusion behind the anterior teeth correction of crossbite. If the answer is yes, then maybe extracts are a better choice for treatment plans. With the support of miniscrew anchorage, Class III malocclusion can be successfully treated with nonextraction methods without subsequent perioral protrusion. In this case, the change in the treatment of Class III malocclusion significantly reduced treatment time and achieved more pleasant profile changes after the correction of anterior crossbite [21].

In Class III cases, the most important trait is a flat posterior occlusal plane [22]. The posterior region's vertical inclination must be corrected by either flattening or steepening the occlusal plane to address these situations [13]. Scientists think that these natural vertical differences happen because of an imbalance in the evolution of the size of the alveolar base and the sizes of all the teeth. This imbalance causes “molar-crowding, “which is made worse when third molars come in and hit each other causing adjacent molars to “squeeze out” [23]. The main goals of MEAW treatment are to rebuild the occlusal plane, get rid of posterior crowding, and straighten back teeth that are angled toward the back of the mouth [24]. These goals can be reached by properly activating the MEAW device. In Class III cases, the MEAW method seems to be helpful for treatment, like making it easier to move all of the lower jaw teeth at once. In this way, the MEAW only detects a small amount of vertical displacement of the posterior teeth during en masse retraction. This shows that it minimizes side effects like extrusive vertical tooth displacement of the anterior teeth, resulting in a higher level of stability compared to the IA. These results are the same as what Chang et al. found in their study [25]. To get the desired results, such as the extrusion of the anterior teeth, vertical elastics must be worn permanently. Otherwise, the tipback bends may result in unwanted intrusion side effects [15].

Miniscrews in the upper alveolar bone are used to provide the necessary anchorage to correct Class III malocclusions. According to the report, the patient was treated with miniscrews in the lower to move the lower teeth apart without the need for the MEAW technique with good results [26, 27]. To straighten the lower teeth without hindering tooth movement, miniscrews in the lower are sometimes placed in the posterior molar area, ascending ramus, or external oblique line of the lower. This is more difficult and may require flap surgery, sometimes causing greater tissue damage. Inserting a miniscrew into the upper is easier, less painful, less uncomfortable, and has a higher success rate. Some clinicians have observed that the cortical bone of the mandible is thicker than that of the maxilla, causing a higher insertion torque and thus a higher rate of failure of attachment to the mandible. In this case, we used miniscrew 1.6 × 10 mm (OrthAnchor, Osstem, Bracket Head, Korea) placed at the buccal region between the second premolar and first molar of the maxillary. The distance between the buccal roots between the second premolar and the first molar, the screw placement location in this case, is the most common location for miniscrew placement to consider for solution surgery because this area is often quite large [28, 29].

Furthermore, the force exerted by the Class III elastic from the first mandible was oriented in a way that made the maxillary miniscrew appropriate for advancing the mandibular anterior teeth. Consequently, this may be advantageous for correcting the crossbite and occlusal plane. Consequently, putting a small screw in the higher region was considered an option, particularly when some patients had challenges in inserting the screw into the lower region. Individuals may effortlessly apply and replace elastic bands with little pain and a high level of adherence.

However, when using the camouflage approach to treat adolescents, it is important to regularly check residual growth to assess the potential for deterioration of mandibular prognathism or facial asymmetry. Regular evaluation is also necessary to ensure long-term stability.

## 4. Conclusion

Class III skeletal malocclusion may be well concealed using MEAW, which combines TADs with modified Class III elastics for camouflage treatment. The mandible can be kept from rotating clockwise, and the upper incisors can be kept from tilting any farther. The MEAW technique, in conjunction with boosted Class III elastics, proved to be an effective treatment option, especially for crossbite tendencies.

## Figures and Tables

**Table 1:** Cephalometric analysis of pretreatment and post-treatment.

**Measurements**	**Norm (SD)**	**Initial**	**Post-treatment**
SNA	81.08 (3.7)	84.75	85.44
SNB	79.17 (3.8)	89.36	84.87
ANB	2.46 (1.8)	−4.61	0.57
Wits appraisal	−0.33 (2.7)	−8.15	−2.96
Facial axis	88.1 (2)	86.75	85.64
*y*-axis	59 (6)	60.05	62.06
Lower anterior face height	65 (5)	58.75	62.9
Mandibular plane angle (Go-Gn to SN)	32 (4)	37.01	38.57
Facial angle	87.8 (3.5)	93.33	92.08
A–B to mandibular plane	69.3 (2.5)	52.39	54.49
ANS-Xi-Pm	47 (4)	48.03	50.26
ODI	72.15 (5.5)	55.19	56
Combination factor	157.9 (6.5)	155.51	149.7
APDI	85.74 (4)	100.32	93.7
Overbite	2 (2)	0.06	1.48
Overjet	2 (2)	−0.47	2.56
U1 to FH	113.8 (6.4)	121.32	120.16
U1 to NA (mm)	4 (3)	6.23	5.27
Interincisal angle	128 (5.3)	124.06	131.91
L1 to A-Pog (mm)	1 (2)	6.8	3.87
L1 to mandibular plane angle	92 (5)	84.54	74.61
Upper molar to PtV	21.1 (3)	14.73	15.51
Nasolabial angle	95 (5)	82.63	78.67
Lower lip to E-plane	0 (2)	3.07	1.63
Upper lip to E-plane	0 (2)	−1.66	−1.59

## Data Availability

Data is available on request from the corresponding author, Lam Nguyen Le (lenguyenlam@ctump.edu.vn).
